# A Course of Clinical Lectures, Delivered at the Hôtel Dieu, Paris, for the Session 1842-’43

**Published:** 1843-07-22

**Authors:** A. F. Chomel


### THE MEDICAL EXAMINER,

### And Retrospect of the Medical Sciences.

Vol. VI.] PHILADELPHIA, SATURDAY, JULY 22, 1843. [No. 14.

### A COURSE OF CLINICAL LECTURES,

*Delivered at the Hôtel Dieu, Paris, for the Session 1842–'43.*

BY A. F. CHOMEL, M.D.

### LECTURE IX.

### RESUMÉ OF THE ANNUAL CLINIC.

(Continued.)

*Typhoid Fever—Pneumonia—Pleurisy.*

Independently of the forms of typhoid fever which we considered in our last lecture, it sometimes occurs in a simple form, without marked predominance of any of the order of symptoms of which we have spoken, without any complication, and without the train of grave accidents which so often accompany the other varieties. This form is the most benign, and most happily the most frequent; for out of one hundred cases it occurred sixty-six times. In the simple or common variety hygienic means have a peculiar efficacy, and really constitute the foundation of the treatment, for our therapeutic measures are almost limited to acidulated drinks, to enemata, and to cataplasms upon the abdomen. Our treatment is in fact nearly expectant. It sometimes happens, however, that at the commencement there are signs of plethora, without any marked inflammatory symptoms. A bleeding or two in such cases may be of advantage. Evacuants can be employed with equal advantage, to modify the progress of the disease, and after having obeyed these indications, you continue to the termination of the disease, the use of demulcent drinks, and hygienic measures, unless other indications should occur from the appearance of other symptoms. Thus, we may have cephalalgia, which it may be necessary to treat by the application of a few leeches behind the ears, and cold compresses to the head. Epistaxis, in general, does not demand any treatment; there are, however, some cases where it is necessary to arrest the flow of blood, which is too abundant, and thus prevent a debility, which might prove fatal. For this purpose you would have recourse to cold acidulated drinks, and cold compresses on the forehead; and should it be rebellious you would resort to the tampon, and plug up the nasal fossa.

The condition of the abdomen claims your particular attention. Formerly practitioners administered purgatives in spite of diarrhœa; now-a-days they are much more reserved on this point. It is generally thought now, that it is not prudent to purge when the intestines are the seat of active irritation from an abundant diarrhœa, from the fear of augmenting the irritation. In this respect it is necessary to proceed with great caution, before deciding. It does not suffice, because some physicians believe that they have discovered its utility; a series of experiments, on a much greater scale, is necessary to determine the degree of this utility. Thus Dr. Louis, who has experimented with this treatment, has obtained some happy results; but these results are so few that they are not sufficient, to my eyes, to warrant any favourable conclusion. At the time Dr. Louis made his experiments, I also tried the same treatment, and chose, for this purpose, four patients, who were subjected to the use of purgatives. Of these four patients three died. This result was so discouraging that I discontinued the experiment. I do not wish to form any conclusion from this small number of facts, but I think that they should at least suggest prudence. When, then, diarrhœa to any degree exists, I do not think the administration of purgatives advisable, for they seem to me, in these cases, opposed to all the principles of sound therapeutics, and I prefer abstaining from them. In such cases I resort to opium, as recommended by Dr. Louis.

Lately a very distinguished physician has recommended the sulphate of quinine, as very efficacious in the treatment of typhoid fever. I have not experimented with this medicine, because after the most generally received ideas in therapeutics, and after the known action of this agent, it seems to me difficult to conceive of its utility in this disease. This medicine has two actions, the one anti-periodic, the other tonic. The first is all-efficacious in periodic maladies, which is not the type of typhoid fever, which is evidently continued. Sometimes, however, you observe in the course of this fever, chills with a sort of exacerbation of the general symptoms; in these cases the sulphate of quinine may be of some utility; but this paroxysmal form is purely accidental, and happens too rarely to make it the indication of a general medication. We reserve, then, this agent for special and exceptional cases, which present this remitting form. As to the second mode of action of the sulphate of quinine—its tonic property—I have already pointed out in what cases it can be useful, and it is for this reason that we should be careful how we employ it in ordinary cases, where it can be much more injurious than useful, especially when there are inflammatory symptoms.

When there is delirium, or a comatose state, I have already indicated what was to be done—local bleedings, if there are signs of cerebral congestion, baths, purgatives, revulsives to the inferior extremities, &c.,—such are the general means employed in similar circumstances. Should intestinal hæmorrhage occur, what is to be done? This is a serious accident which it is necessary to combat promptly, for it can only serve to prostrate still more the organism already enfeebled. Cold enemeta should be employed. When the hæmorrhage is from the superior part of the intestine this method will not succeed, and we must have recourse to astringents, as sulphuric lemonade, rhatany, catechu, &c. Finally, intestinal perforation may occur as a consequence of the fall of the eschars of the intestines. Formerly this malady was considered as necessarily mortal; death is in fact the most common result; it sometimes happens, however, that patient's recover. So soon as you can diagnosticate intestinal perforation, the patient must be subjected to entire rest, stop all drinks, and in their place merely keep the mouth of the patient moist with juice of orange. By this means you may hope to prevent all motion in the intestines, and by it the effusion of new matter into the peritoneal cavity. To this you will add large doses of opium, with the double object of blunting the intestinal irritation, and producing a kind of narcotism, proper to procure a favourable calm.

I shall consider under the same head, inflammations of the lung and those of the pleura, as we more frequently meet with pleuro-pneumonias, than pure and simple pneumonias. Afterwards I shall notice those cases of simple pleurisy that exist independently of any alteration of the pulmonary parenchyma.

We have had, in the course of the year, eighty-one cases of pleuro-pneumonia. Of these forty-nine were during the summer semestre, and thirty-two during the winter one. In this number we have taken into account only primitive pneumonias, considered as the principal affection, reserving for the period when we treat of other diseases, those cases of pneumonia which were developed consecutively to other affections already existing—as diseases of the heart, stomach, brain, &c. Out of the eighty-one cases we had twelve deaths, that is a little more than one in nine. Thus the mortality has been, as you see, very slight, and much less than it was the preceding years. There was some difference in the mortality during the winter and summer months, being about one in eight in winter, and one in six in summer. This fact agrees with the observation that we have made for some years past, and above all with the results of the last eleven years, which I shall present to you presently. In fact, pneumonias are more frequent in summer, and particularly in the spring, periods, too, at which they present a very considerable degree of gravity. On taking the general mean of the eleven years of which we have kept an exact account, we find precisely the same proportion in the mortality.

Considered relatively to the sexes, the mortality presents also different proportions—being one in eight for men, and one in six for women. This fact is the more remarkable inasmuch as the disease is more common among men than women, and we have had a much larger number of cases in the men's wards than in the women's, although the number of beds in the latter is much more considerable than in the former.

This relative frequency of pneumonia among males is generally explained on the ground of their greater active vitality, and to the nature of the labors to which they are habitually exposed, being much more rude than those of the other sex. You can, to a certain degree, explain also the less degree of mortality among the men, to the greater vigour of their constitutions, and their greater aptitude to resist the progress of inflammation. This difference is still more striking with regard to age. In eleven years we have had sixty-seven cases of pneumonia, in individuals aged from fifteen to twenty years, (at the Hôtel-Dieu no patients are received under 15,) out of this number we have not had one case of death; all recovered, notwithstanding the severity of the disease in many cases. We lately had in our wards a young man, who had pneumonia, followed by parotiditis with gangrene and paralysis of the facial nerve. In spite of these very grave complications he was discharged cured. In proportion as patients are advanced in life, inflammation of the lungs becomes more and more grave, and more frequently mortal. Thus in subjects from twenty to forty years of age, the mortality this year was one in ten. Out of forty-five cases which we had at this period of life we lost three. From forty to sixty years we had twenty-two cases of pneumonia and four deaths, a little less than one in five. In the eleven preceding years the mortality was for this period one in five. Finally, beyond sixty years of age we have had seven cases and five deaths, that is to say more than two-thirds, which constitutes a frightful proportion, and one much greater than the mean of the previous eleven years, where it was little more than one-half.

From these statistics it results that when you have a pneumonia in a subject aged from fifteen to thirty, the prognosis should be favourable; at least, the chances are strongly in favour of recovery. But in proportion as you approach sixty years, and above all when you go beyond that age, you have always to dread a fatal termination.

Pneumonia presents also a great deal of interest in relation to the duplicity of the organ it affects. This year we have had three cases of double pneumonia, and of this number two died. In the preceding years, where we have kept any account, there was a mortality of one half, a little less consequently than that of the current year.

The results of this disease vary also, in respect to the seat of the disease, whether in the right or left lung, and whether at the summit or base of the lung; it results from our observations, that the mortality is greater for pneumonia of the right lung, and for that of the summit. Thus in the cases of the eleven preceding years the mortality was one in six for pneumonias of the right side, and one in eleven only for pneumonias of the left side. This year it was one in six for the right side, and one in fifteen for the left side, a still greater difference in favour of these last than in the preceding years. The cause of this difference is difficult enough to determine. You may perhaps say that pneumonia, being more grave in proportion as it occupies a greater surface, that of the right side will be more grave, on account of the greater volume of the right lung. This is but an hypothesis, but an hypothesis which is perhaps not without foundation.

As regards the seat of pneumonia, we have had six cases of pneumonia of the summit, seventy-three cases of pneumonia of the base, and two cases where the seat of the disease was the middle portion of the lung.

It is well known that pneumonias of the summit are more serious than those of the base. What is the cause of this? Is it because they are often connected with the existence of tubercles in this portion of the lung! This is possible. This coincidence in fact is sometimes presented, but very frequently you can discover no trace of tubercles in the summit of the lung, which is the seat oi the phlegmasia; so that the true cause which makes pneumonias of the summit more fatal than those of the base, remains yet to be discovered.

Pneumonia is interesting in connection with prognosis, as it is a first or second attack, single or double, simple or complicated. I have had occasion very often to state to you this general fact—that a phlegmasia is less intense as it is repeated, and reproduced often in the same organ, to the extent that these phlegmasias are sometimes resolved at the end of several days, when they have been reproduced several times. This is a principle of pathology, which is the result of repeated observation upon a great number of cases. Thus how often have we seen erysipelases of the face, for example, so grave and alarming, when they are developed for the first time, lose their intensity, and become very easy to cure when they have occurred several times in the same individual. It is the same with pneumonia. This principle has not been falsified by the facts which we have observed this year. Thus in twenty-five pneumonias, not first attacks, there were only three deaths, whilst the mortality was proportionately much greater in cases of primitive pneumonia. These observations should be followed up by others, and I am convinced that they will arrive at results still more satisfactory.

We have studied the symptoms of interest which pneumonia presents, whether in diagnosis or prognosis. Thus we have noted the sputa, and its appearance. In five cases this year we had the characteristic rust-coloured sputa; in seventeen cases it was colourless, and in other cases it was wanting altogether, although there was no doubt of the existence of the disease. The mortality was greatest in those cases where the sputa was sanguinolent. The frequency of the pulse afforded us very important results for prognosis. Thus in forty-three patients suffering from pneumonia, we had a pulse varying from 80 to 112, and in this number the mortality was one in thirteen. On the contrary, in sixteen cases, where the pulse gave a minimum of 120 pulsations, the mortality was one in three. Hence, whenever we encounter, in a pneumonia patient, a pulse beyond 120 beats we can give an unfavourable prognosis with great certainty. The importance of these statistics, in regard to practice, you will readily comprehend.

